# Synthesis, Structures and Luminescence Properties of Metal-Organic Frameworks Based on Lithium-Lanthanide and Terephthalate

**DOI:** 10.3390/polym8030086

**Published:** 2016-03-16

**Authors:** Mohammed S. M. Abdelbaky, Zakariae Amghouz, Santiago García-Granda, José R. García

**Affiliations:** 1 Departamentos de Química Física y Analítica y Química Orgánica e Inorgánica, Universidad de Oviedo-CINN, 33006 Oviedo, Spain; saidmohammed.uo@uniovi.es (M.S.M.A.); sgg@uniovi.es (S.G.-G.); jrgm@uniovi.es (J.R.G.); 2 Servicios Científico-Técnicos, Universidad de Oviedo-CINN, 33006 Oviedo, Spain

**Keywords:** lanthanide-organic frameworks, dicarboxylate, hydrothermal, crystal structure, topology, photoluminescence

## Abstract

Metal-organic frameworks assembled from Ln(III), Li(I) and rigid dicarboxylate ligand, formulated as [LiLn(BDC)_2_(H_2_O)·2(H_2_O)] (**MS1-6**,**7a**) and [LiTb(BDC)_2_] (**MS7b**) (Ln = Tb, Dy, Ho, Er, Yb, Y_0.96_Eu_0.04_, Y_0.93_Tb_0.07_, and H_2_BDC = terephthalic acid), were obtained under hydrothermal conditions. The isostructural **MS1-6** crystallize in monoclinic *P*2_1_/c space group. While, in the case of Tb^3+^ a mixture of at least two phases was obtained, the former one (**MS7a**) and a new monoclinic *C*2/c phase (**MS7b**). All compounds have been studied by single-crystal and powder X-ray diffraction, thermal analyses (TGA), vibrational spectroscopy (FTIR), and scanning electron microscopy (SEM-EDX). The structures of **MS1-6** and **MS7a** are built up of inorganic-organic hybrid chains. These chains constructed from unusual four-membered rings, are formed by edge- and vertex-shared {LnO_8_} and {LiO_4_} polyhedra through oxygen atoms O3 (vertex) and O6-O7 (edge). Each chain is cross-linked to six neighboring chains through six terephthalate bridges. While, the structure of **MS7b** is constructed from double inorganic chains, and each chain is, in turn, related symmetrically to the adjacent one through the *c* glide plane. These chains are formed by infinitely alternating {LiO_4_} and {TbO_8_} polyhedra through (O2-O3) edges to create Tb–O–Li connectivity along the *c*-axis. Both **MS1-6,7a** and **MS7b** structures possess a 3D framework with 1D trigonal channels running along the *a* and *c* axes, containing water molecules and anhydrous, respectively. Topological studies revealed that **MS1-6** and **MS7a** have a new 2-nodal 3,10-c net, while **MS7b** generates a 3D net with unusual β-Sn topology. The photoluminescence properties Eu- and Tb-doped compounds (**MS5-6**) are also investigated, exhibiting strong red and green light emissions, respectively, which are attributed to the efficient energy transfer process from the BDC ligand to Eu^3+^ and Tb^3+^.

## 1. Introduction

Metal-organic frameworks (MOFs), as an important class of advanced functional materials, have received extensive attention due to their great potential application in various research areas, such as gas adsorption/storage [1,2,3,4,5], liquid separation [6], drug delivery [4,7], and heterogeneous catalysis [8,9,10]. Among different classes of MOFs, lanthanide MOFs (LnMOFs) [11,12,13,14,15], which can be synthesized using a wide range of lanthanide cations and organic ligands, have been investigated in the fields of coordination chemistry, inorganic chemistry, and material chemistry, not only for their diverse architectures due to high coordination numbers and large ionic radii of lanthanide cations, but also for the potential applications in the field of luminescence [13,14,15,16,17,18,19,20,21], because of their increased brightness and emission quantum yield [22,23], and also in magnetism [11,24,25,26,27,28]. To obtain functional MOFs, considerable efforts have been made and tremendous progress has been achieved. However, challenges in crystal engineering still remain for controlling the chemical composition and dimensionality. It is well-known that the crystal structure is generally driven by the coordination mode of the metal center, solvent system, temperature, pH, and so on [29,30,31,32]. Furthermore, the geometry and nature of the organic ligands play an important role in determining the final structures and topologies [33]. As an important class of organic ligands, those containing the carboxyl groups that have been applied in the preparation of LnMOFs due to the affinity of lanthanides cations to carboxylate oxygen atoms. In particular, we focused our research on the dianion organic linker 1,4-benzenedicarboxylic acid (BDC), due to its structural rigidity, a diversity of coordination geometries, and the possibility to facilitate the formation of structures with large voids [34,35]. Alkali-based MOFs [36] are not extensively explored, in comparison with transition metals or lanthanide-based MOFs, despite the incorporation of alkali cations into MOFs being an interesting development of MOF chemistry, through exploitation of their various coordination modes, low polarizability, and unique affinity for basic molecules [37,38]. Among these compounds, the lithium-based MOFs [39,40,41,42,43,44,45,46] are attracting particular interest due to recent experimental and theoretical studies showing enhanced H_2_ uptake in lithium doped MOFs [47,48,49,50,51,52,53,54]. Moreover, they are promising candidates for replacing the conventional electrode in Li-ion batteries, exhibiting a high reversible specific capacity and excellent cyclability [55,56,57,58]. Many studies have been carried out using the hydrothermal route to prepare Ln–BDC [20,59,60,61] or alkali–BDC [62,63,64,65] based frameworks, while the first example of MOFs assembled from Li–Ln–BDC has been recently reported by us [12].

Inspired by this previous work and in continuation of our ongoing studies on the design of novel alkali-lanthanide hetero-MOFs, herein we report the full structural characterization of novel lithium-lanthanide-containing MOFs, formulated as [LiLn(BDC)_2_(H_2_O)**·**2(H_2_O)] (Ln = Dy (**MS1**), Ho (**MS2**), Er (**MS3**), Yb (**MS4**), Y_0.96_Eu_0.04_ (**MS5**), and Y_0.93_Tb_0.07_ (**MS6**)) and [LiTb(BDC)_2_] (**MS7b**). Their luminescence properties have been investigated.

## 2. Experimental Section

### 2.1. Hydrothermal Synthesis

[LiLn(BDC)_2_(H_2_O)]·2(H_2_O) (**MS1-4** and **MS7**) were synthesized under hydrothermal conditions. In a typical synthesis, 0.08 g (0.5 mmol) of terephthalic acid (H_2_BDC), 0.14 g (3.5 mmol) of LiOH·H_2_O, and (1 mmol) of LnCl_3_·6H_2_O (Tb: 37 g; Dy: 0.38 g; Ho: 0.38 g; Er: 0.38 g; Yb: 0.39 g) were dissolved in a mixture of distilled water (5 mL) and ethanol (5 mL). [Eu_0.04_Y_0.96_(BDC)_2_(H_2_O)]·2H_2_O (**MS5**) and [Tb_0.07_Y_0.93_(BDC)_2_(H_2_O)]·2H_2_O (**MS6**) have been obtained following the same procedures as described above, by using solutions of Y:Ln with a molar ratio of 19:1 previously prepared by dissolving: 0.29 g (0.95 mmol) of YCl_3_·6H_2_O and 0.03 g (0.05 mmol) of EuCl_3_·6H_2_O or TbCl_3_·6H_2_O for **MS5** and **MS6**, respectively, in a mixture of ethanol (5 mL) and distilled water (5 mL). The both solutions have been stirred for 24 h. The synthesis of all compounds has been done in presence of tartaric acid and an amount of 1 mmol (0.21 g) was necessary to obtain these compounds. In all cases the reaction mixture was stirred at room temperature to homogeneity and then placed in a Teflon-lined stainless vessel (40 mL) and heated to 180 °C for three days under autogenous pressure and, afterwards, cooled to room temperature. The resulting products were filtered off, washed thoroughly with distilled water, and finally air-dried at room temperature. Yield: 27 mg, *ca.* 12% for all compounds based on Ln. A crystalline phase of parallelepiped-like single-crystals and microcrystalline powder was obtained in the case of **MS1-6** while, for **MS7**, a mixture of plate- and parallelepiped-like crystals and a microcrystalline powder was obtained.

### 2.2. Single-Crystal X-Ray Diffraction Studies

Collection was performed at 293 K on an Agilent Gemini CCD diffractometer (Oxford, Oxfordshire, UK), using CuKα radiation. Images were collected at a 55 mm fixed crystal-detector distance, using the oscillation method, with 1° oscillation and variable exposure time per image. The crystal structure was solved by direct methods. The refinement was performed using full-matrix least squares on F^2^. All non-H atoms were anisotropically refined. All H atoms were either geometrically placed riding on their parent atoms or located from the difference Fourier map, with isotropic displacement parameters set to 1.2 times the Ueq of the atoms to which they are attached. Crystallographic calculations were carried out using the following programs: CrysAlis CCD [66] for data collection; CrysAlis RED [67] for cell refinement, data reduction and empirical absorption correction; SHELXS-97 [68] for structure solution; XABS2 [69] for refined absorption correction; SHELXL-97 for structure refinement and prepare materials for publication; PLATON [70,71] for the geometrical calculations; and Diamoned [72] for molecular graphics.

### 2.3. Powder X-Ray Diffraction Studies

**Figure 1:**
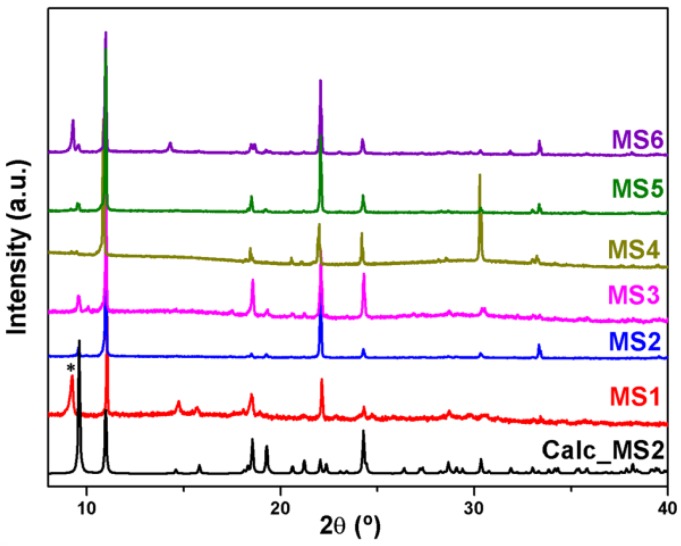
PXRD patterns of **MS1-6** compared with the calculated for **MS2**; (*) shows the peak of an unidentified phase.

**Figure 2:**
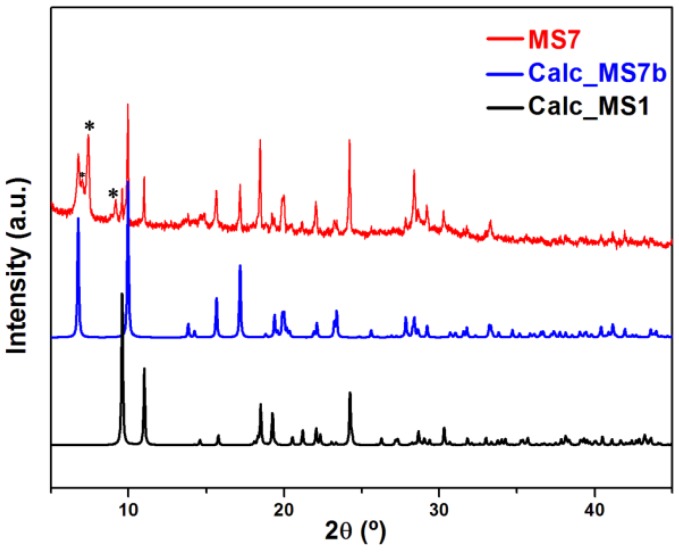
PXRD pattern of **MS7**, compared with the calculated for **MS1** and **MS7b**; (*) shows the peaks of an unidentified phase.

Powder X-ray diffraction patterns were recorded on X’pert Philips diffractometer with CuKα radiation. The samples were gently ground in an agate mortar in order to minimize the preferred orientation. All data were collected at room temperature over the angular 2θ range 4°–60° with a step of 0.01° and a counting time of 1.5 s/step. The PXRD patterns of the compounds were compared with the calculated ones, indicating that the products have been successfully obtained as crystalline phase for **MS1-6** (Figure 1) and as a crystalline mixture composed of at least two phases for **MS7** (Figure 2).

### 2.4. Morphological Characterization

SEM micrographs and X-ray microanalysis (SEM/EDX) were recorded by using JEOL-6610LV scanning electron microscope (Akishima, Tokyo, Japan) operating at 30 kV coupled with an Oxford X-Max microanalysis system (EDX). SEM images (Figure S1) show parallelepiped-like morphology and micropowder for **MS3** sample, while the plate- and parallelepiped-like morphologies along with micropowder were observed in the case of **MS7** sample. EDX provided Y:Eu and Y:Tb quantitative analysis for **MS5** and **MS6** (Figure S2 and Table S1).

### 2.5. Infrared Spectra

The infrared data were collected at room temperature using a FT-IR Bruker Tensor-27 spectrometer (Billerica, MA, USA) from KBr pellets. The spectra were collected over the range 4000–400 cm^−1^ by averaging 15 scans at a maximum resolution of 4 cm^−1^.

### 2.6. Thermal Characterization

A Mettler-Toledo TGA/SDTA851e (Greifensee, Switzerland) was used for the thermal analysis in oxygen dynamic atmosphere (50 mL/min) at a heating rate of 10 °C/min. In this case, *ca*. 10 mg of powder sample was thermally treated, and blank runs were performed.

### 2.7. Photoluminescence Studies

RT excitation and emission spectra and luminescence lifetimes were measured using a standard spectrofluorometer Edinburgh Instruments FLSP920 (Edimburgh, Scotland, UK), having a 450W Xe lamp as the excitation source. The samples were placed between two quartz plates placed at 45° from the incident beam and the detector. The luminescence lifetimes sample was excited using a nanopulsed light source at 260 nm. Luminescence decay curves were recorded using a fast-response MCP-PMT detector in cooled housing. Lifetime was estimated by fitting the decay curve with a mathematical model, according to the general formula *I* = *I*_0_ e ^(−t/tau)^ where tau is the lifetime. The optical images of the sample were obtained, upon excitation at 360–370 nm and detecting the emission over 420 nm, by using an automated optical microscope Olympus CAST2 for transmission and fluorescence studies equipped with a motorized stage and a high-resolution digital camera.

## 3. Results

### 3.1. Description of the Crystal Structures

The series of compounds **MS1-6** are isoreticular and crystallize in the same monoclinic space group *P*2_1_/c as revealed by single-crystal X-ray diffraction studies. While the second phase **MS7b** crystallizes in the monoclinic space group *C*2/c. The crystallographic and structure refinement data are presented in Table 1, whereas Table S2 shows selected bond angles and lengths for **MS1-4** and **MS7b** compounds. Powder X-ray diffraction measurements (Figure 1), confirm that the series **MS1-6** have been successfully obtained as crystalline phases and that the crystals exhibit a strong preferential orientations. However, in case of Tb, the powder X-ray diffraction (Figure 2) indicates that the product of the synthesis consist of at least two identified phases, **MS7a** which belongs to the series **MS1–6** and the second novel phase **MS7b**.

**Figure 3:**
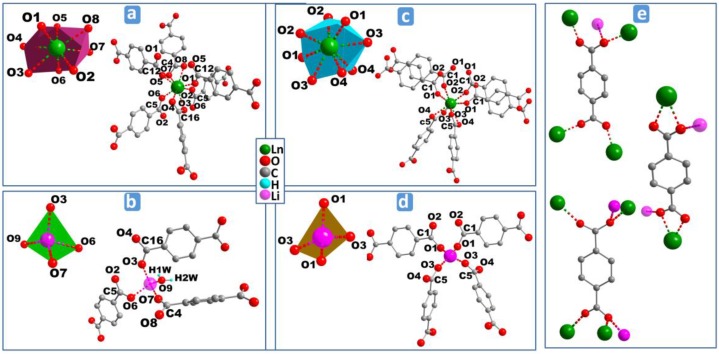
Perspective view of the coordination environments of Ln^3+^ and Li^+^ cations in **MS1–6** (**a**,**b**) and **MS7b** (**c**,**d**) and coordination modes of BDC ligand (**e**).

**Figure 4:**
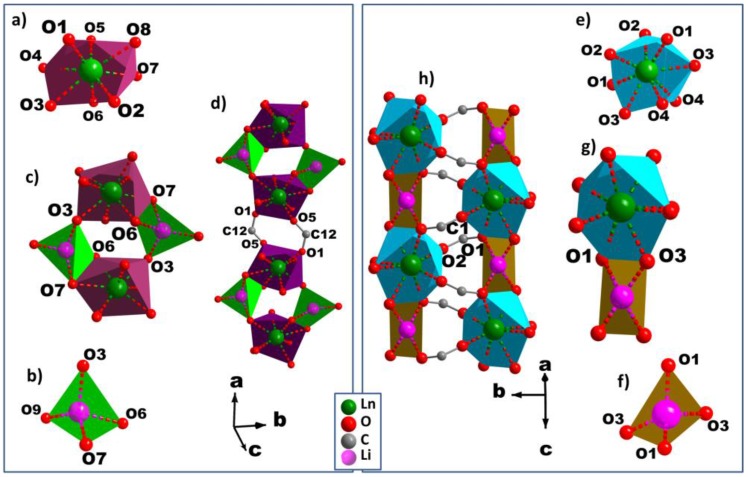
Perspective view of the coordination environments of Ln^3+^ and Li^+^ cations in **MS1–6** (**a**,**b**) and **MS7b** (**e**,**f**); the secondary building unit for **MS1–6** (**c**) and **MS7b** (**g**), and the infinite chains along the *a* and *c* axes for **MS1–6** (**d**) and **MS7b** (**h**).

**Figure 5:**
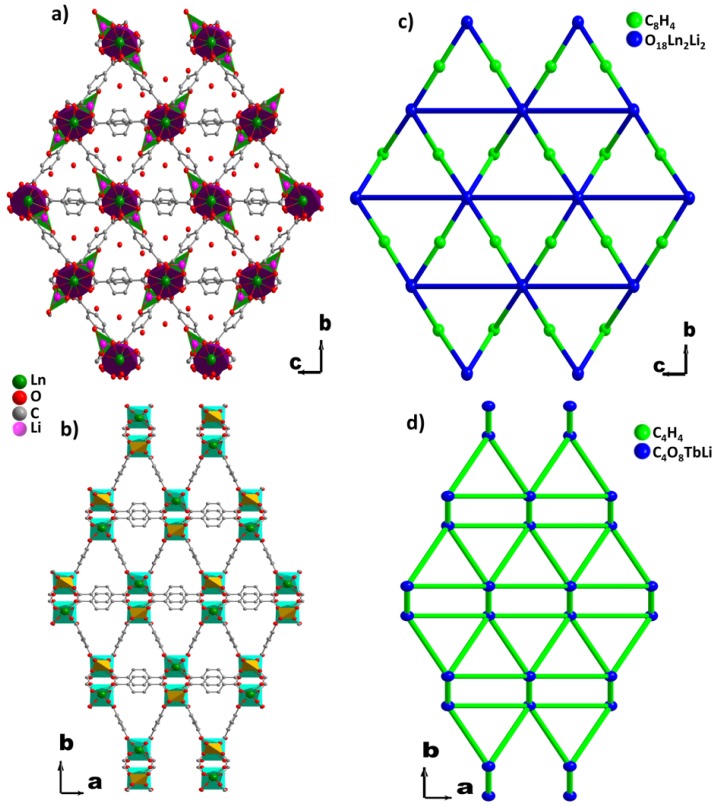
Projection of the structure along the *a* and *c* axes for **MS1–6** (**a**) and **MS7b** (**b**), respectively. Topological representations of **MS1–6** (**c**) and **MS7b** (**d**).

The asymmetric units in case of **MS1–6** (Figure S3a), comprises one Ln^3+^, one Li^+^, one BDC, two half-BDC, one coordinated water molecule, and two non-coordinated water molecules. The Ln^3+^ cation is bonded to eight oxygen atoms, all of them coming from six carboxylate groups which belong to four non-equivalent groups. Its coordination geometry may be described as a distorted bicapped trigonal prism (Figure 3a). Out of the four carboxylate groups surrounding Ln^3+^, which belong to two different carboxylate groups, each of two contribute with one oxygen atom, and the two remaining carboylates act in chelating mode. The eight Ln–O bond distances range from 2.212(2) to 2.473(2) Å, and the average value decreases from 2.368 Å (Dy), 2.362 Å (Ho), 2.348 Å (Er), to 2.334 Å (Yb). Each Li^+^ ion assumes a distorted tetrahedron environment by coordinating to four crystallographically-independent oxygen atoms (Figure 3b). These oxygen atoms come from three independent carboxylate groups and one of water molecules. The Li–O bond distances range from 1.880(2) Å to 2.060(2) Å, with the average values of 1.950 Å (Dy), 1.950 Å (Ho), 1.949 Å (Er), and 1.945 Å (Yb), indicating that the environment of Li^+^ is not affected by the type of Ln^3+^.The 3D structures of **MS1-6** series are built up of inorganic-organic hybrid chains;, each chain is, in turn, crosslinked to six neighboring chains through six terephthalate bridges. These chains constructed from unusual four-membered rings (Figure 4c) are formed by edge- and vertex-shared {LnO_8_} and {LiO_4_} polyhedra (Figure 4a,b) through oxygen atoms O6–O7 (edge) and O3(vertex) (Er, for instance). The isolated four-membered rings stacked along the *a*-axis, are bridged via two equivalent carboxylate groups O1–C12–O5 forming the above mentioned hybrid chains (Figure 4d). The distance between adjacent Ln–Li cations in the chain has an average value of 3.395 Å. The 3D framework with trigonal channels running along the *a*-axis contains the crystallization water molecules (Figure 5a), which are involved in strong hydrogen bonds between each other and with the carboxylate oxygen atoms (Table S3).

In the case of **MS7b**, the asymmetric unit (Figure S3b) consists of one Tb^3+^, one Li^+^, and two half-BDC. Tb^3+^ cation shows a distorted square antiprism geometry (Figure 3c) with the average Tb–O bond distances of 2.361 Å. While Li^+^ cation exhibts a similar distorted tetrahedron coordination polyhedron, like Li^+^ in the series **MS1–6**. Out of the four carboxylate groups surrounding the Li^+^ (Figure 3d), which belong to two independent carboxylate groups O1–C1–O2 and O3–C5–O4, each of two contribute with one oxygen atom O1 and O3, where the average Li–O bond distances is 1.943 Å. The structure of **MS7** is constructed from double inorganic chains running along the *c*-axis. Each chain is formed from edge (O2–O3) shared {LiO_4_} and {TbO_8_} polyhedra (Figure 4e–g). The {LiO_4_} and {TbO_8_} polyhedra alternate infinitely in a chain to create Tb–O–Li connectivity along the *c* direction (Figure 4h), with the Li–Ln distance between adjacent cations in the chain of 3.3765(6) Å. Each chain is symmetrically related to the neighbor one through *c* glide plane. These chains are connected via carboxylate group O1–C1–O2 along *c*-axis, forming the inorganic-organic double chains (Figure 4h). These hybrid double chains are, in turn, linked to six neighboring units via six BDC bridges, forming the 3D framework with empty trigonal channels running along the *c-*axis (Figure 5b).

The coordination modes of BDC anions, found in both **MS1–6** and **MS7b**, are shown in Figure 3e. Each carboxylate group belongs to one of the three modes: (i) groups coordinating one oxygen atom to one Ln^3+^ cation where the other oxygen is bonded in bridging mode between other Ln^3+^ and Li^+^ cations; (ii) groups bridging two adjacent Ln^3+^ cations; or (iii) bonded to one Ln^3+^ cation via two oxygen atoms, and in bridging mode between Ln^3+^ and Li^+^ cations through one of these oxygen atoms. The coordination modes (i) and (iii) have been found in both **MS1–6** and **MS7b**, while mode (ii) has only been found in **MS1–6**.

The topology of **MS1–6** and **MS7b** have been analyzed with the TOPOS software [73], reducing the structure to a simpler node-and-linker net [74]. The framework of **MS1–6** reveals a novel binodal net topology (Figure 5c), simplified as a 2-nodal 3,10-c net with the point symbol of {4.5^2}- 2{4^14.5^10.6^18.7.8^2}. The framework of **MS7b** exhibits unusual β-Sn topology with a uninodal 6-connected net with the point symbol of {4^8.5^4.6^3} (Figure 5d).

### 3.2. IR Analysis

**Figure 6:**
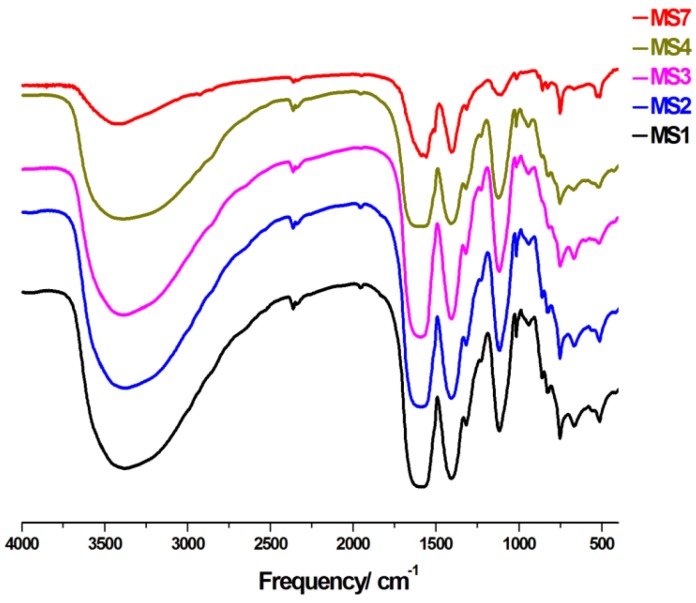
IR spectra of **MS1–4** and **MS7**.

The IR spectra of **MS1–4** and **MS7** are shown in Figure 6. The broad band observed in the 3710–2820 cm^−1^ region is assigned to O–H stretching vibrations of coordinated and uncoordinated water molecules, where in the case of **MS7**, this band belongs to the hydrated phase **MS7a**. The characteristic bands for the antisymmetric *ν*_asym(C═O)_ and symmetric *ν*_sym(C═O)_ vibrations appear at *ca.* 1589 cm^−1^ and 1409 cm^−1^, respectively. The difference between the bands (Δ*ν*_asym(COO)–sym(COO)_ = 180 cm^−1^) confirms the bridging mode of the carboxylate groups found in these compounds.

### 3.3. Thermal Analysis

**Figure 7:**
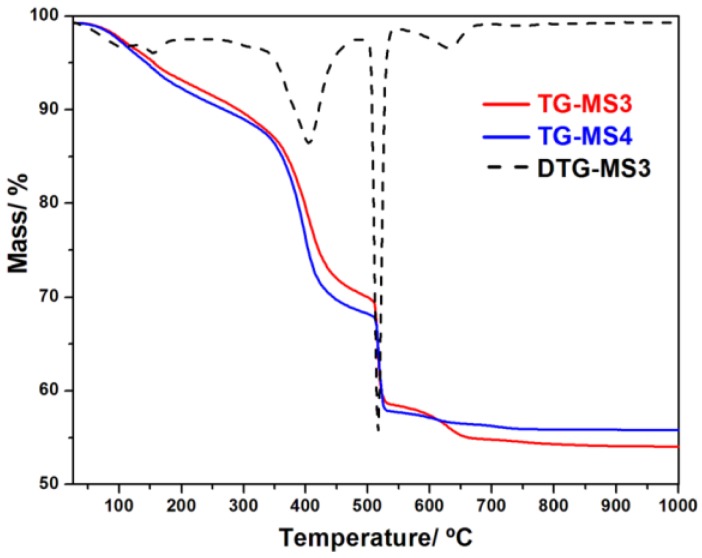
TGA curves of **MS3** (Er) and **MS4** (Yb).

The thermal stability of **MS1–6** compounds was investigated by thermogravimetric analysis (TGA/DTG). Figure 7 shows the thermal behavior of **MS3** and **MS4**, while the thermal behavior of **MS1**, **MS2**, **MS5**, and **MS6** is shown in Figure S4. All compounds show a similar thermal behavior. The first and second weight loss in the range 25–200 °C correspond to the loss of the two guest water molecules. The third weight loss between 260 and 330 °C is attributed to the loss of the coordinated water molecule. The forth weight loss from 330 to 470 °C corresponds to the beginning of the decomposition of BDC ligand, while in the fifth step between 480 and 550 °C the complete oxidation of the BDC is taking place. Further weight loss above 550 °C is expected to be due to the evacuation of trapped carbonaceous residual species.

### 3.4. Potoluminescence Properties

**Figure 8:**
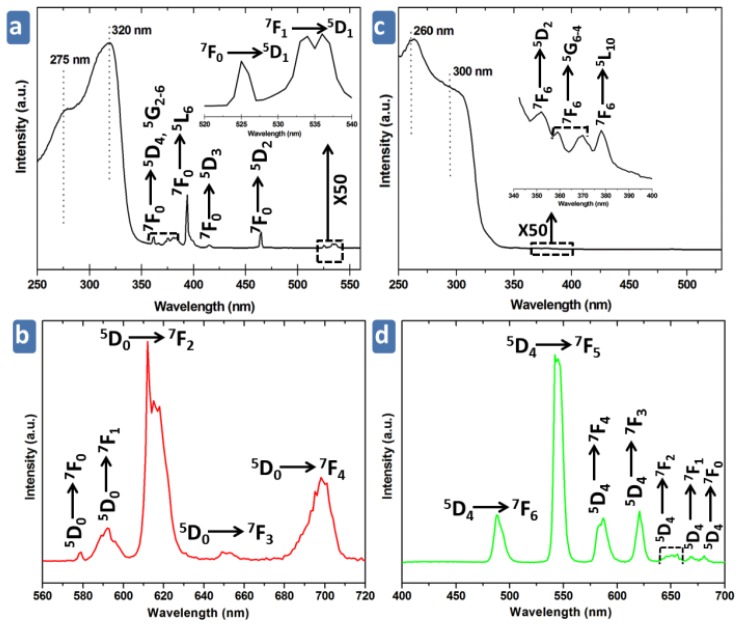

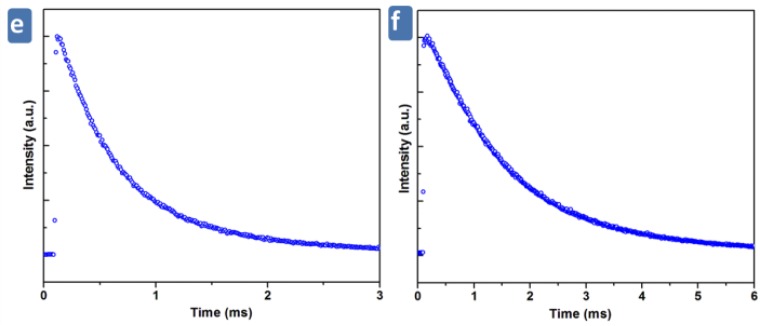
Excitation spectra for **MS5** (**a**) and **MS6** (**c**) detecting luminescence at 615 and 545 nm, respectively. Emission spectra for **MS5** (**b**) and **MS6** (**d**) upon excitation at 260 and 275 nm. Emission decay curves excited at 260 nm and monitored at 616 and 545 nm for **MS5** (**e**) and **MS6** (**f**), respectively.

**Figure 9:**
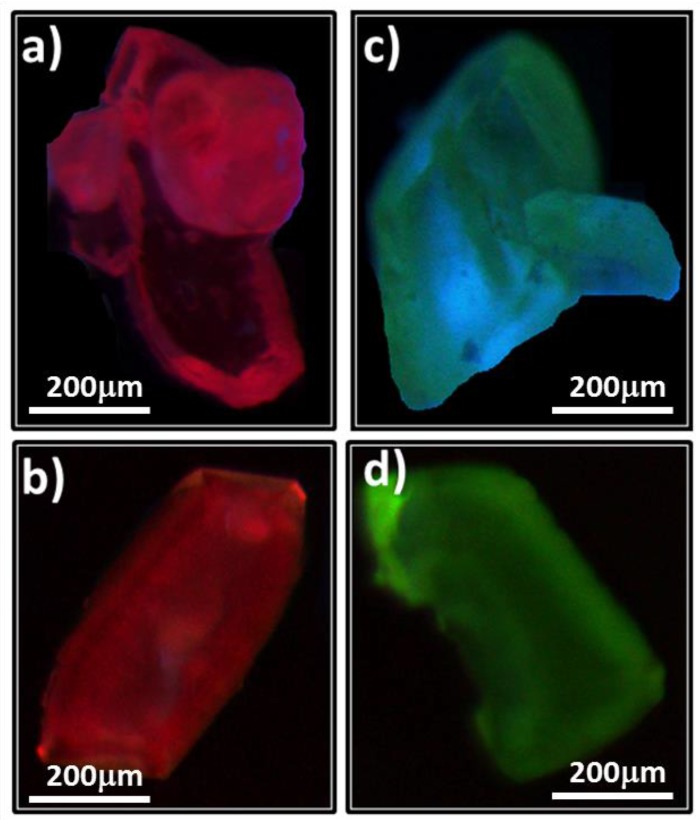
Optical microscopic images under UV light of single crystals of Eu-doped compound **MS5** (**a**,**b**) and Tb-doped compound **MS6** (**c**,**d**).

Taking into account the excellent luminescent properties of Eu^3+^ and Tb^3+^ cations, the doped compounds **MS5** and **MS6** have been prepared as described above. The photoluminescence properties of **MS5** and **MS6** were investigated at room temperature. The RT luminescence and excitation spectra of **MS5** and **MS6** are shown in Figure 8. Moreover, selected single-crystals of **MS5** and **MS6** have been examined by optical microscopy under UV light, and they exhibit strong red and blue-green light emissions, respectively, as illustrated in Figure 9. The both bluish and green light emissions observed in the case of **MS6** are related to the variation of Tb^3+^ content in the crystals, which indicates that the emission could be tuned from green to blue by changing the Tb^3+^ content. The excitation spectrum of **MS5**, monitored within the Eu^3+ 5^D_0_ → ^7^F_2_ transition, shows a large band with two maxima at *ca.* 275 nm and 320 nm are attributed to the excited state of BDC ligand, and also a series of peaks attributed to the electronic transition from the ground states ^7^F_0,1_ to the excited states ^5^D_4-1_, ^5^G_2-6_, and ^5^L_6_ according to the Dieke’s diagram [75]. Its emission spectrum, upon excitation at 275 nm, exhibits the characteristic emission lines for Eu^3+^ cations centered at 580, 590, 615, 651, and 698 nm, which result from deactivation of the ^5^D_0_ excited state down to the ^7^F_4-0_ ground states (Figure 8b). The most intense emission peak, centered at 615 nm and corresponding to the hypersensitive ^5^D_0_ → ^7^F_2_ transition, implies red emission light of Eu^3+^ with a lifetime τ = 0.84 ± 0.01 ms (Figure 8e). The excitation spectrum of **MS6**, monitored within the Tb^3+ 5^D_4_ → ^7^F_5_ transition, exhibits a large band (maxima at *ca.* 260 and 300 nm) assigned to BDC ligand and a series of lines assigned to the transition from the ground stats ^7^F_6_ to the excited stats ^5^D_2_, ^5^G_6-4_, and ^5^L_10_ [76] (Figure 8c). Its emission spectrum upon excitation at 260 nm exhibits the characteristic emission peaks for Tb^3+^ cation centered at 490, 545, 585, 620, 650, 668, and 681 nm, which result from deactivation of the ^5^D_4_ excited state down to the ^7^F_6-0_ ground state multipelts (Figure 8d). The most striking green luminescence centered at 545 nm corresponds to the hypersensitive transition^5^D_4_ → ^7^F_5_ with a lifetime τ = 1.37 ± 0.01 ms (Figure 8f). Furthermore, in both **MS5** and **MS6** excitation spectra, the low intensity of the intra ^4^f_6_ and ^4^f_8_ peaks with respect to the strong bands at wavelengths smaller than 350 nm point out that the Eu^3+^ and Tb^3+^ cation sensitization, via charge transfer from the BDC ligand, is more efficient when compared to direct 4f excitations.

Moreover, PL properties of **MS3** were also studied (Figure S5). The excitation spectrum monitored around Er^3+ 4^I_15/2_ → ^4^S_3/2_ transition exhibits an intense peak at 376 nm, which is attributed to the electronic transition from ^4^I_15/2_ ground state to the excited state ^3^G_11/2_. Its emission spectrum upon excitation at 370 nm shows the characteristic emission peaks of Er^3+^ located at 414, 437,492, 582, and 557 nm corresponding to transitions from ^2^G_9/2_, ^2^F_9,5,7/2_, ^2^H_9,11/2_, and ^4^S_3/2_ down to ^4^I_15/2_[14,77].

## 4. Conclusions

In summary, we have synthesized and characterized novel MOFs assembled from Li^+^, Ln^3+^, and rigid dicarboxylate ligands, formulated as [LiLn(BDC)_2_(H_2_O)·2(H_2_O)] (MS1–4) (where Ln = Dy, Ho, Er, and Yb) and [LiTb(BDC)_2_] (**MS7b**). Their structures were determined by single X-ray diffraction are based on unusual four-membered rings {Li_2_Ln_2_O_18_} and double inorganic chains constructed from {LiTbO_10_} dimeric units, and both exhibit a 3D framework with 1D trigonal channels running along the *a* and *c* axes, containing guest water molecules and anhydrous, respectively. Photoluminescence properties of **MS5** and **MS6** have been studied showing strong red and green light emissions, attributed to the effective Eu^3+^ and Tb^3+^ sensitization via ligands, with a lifetime of 0.84 ± 0.01 ms and 1.37 ± 0.01 ms, respectively.

## Figures and Tables

**Table 1:** Crystallographic data for **MS1–4** and **MS7b**.

Identification Code	MS1	MS2	MS3	MS4	MS7b
**Empirical formula**	C_16_H_14_O_11_DyLi	C_16_H_14_O_11_HoLi	C_16_H_14_O_11_ErLi	C_16_H_14_O_11_YbLi	C_16_H_8_O_8_TbLi
**Formula weight/g·mol^−1^**	551.71	554.14	556.47	562.25	494.09
**Temperature/K**	293(2)	296(2)	293(2)	293(2)	293(2)
**Wave length**	0.71073	0.71073	0.71073	0.71073	0.71073
**Crystal system**	Monoclinic	Monoclinic	Monoclinic	Monoclinic	Monoclinic
**Space group**	*P*2_1_/c	*P*2_1_/c	*P*2_1_/c	*P*2_1_/c	*C*2/c
**Unit cell dimensions**					
**a(Å)**	11.6769(4)	10.1727(7)	10.1678(3)	10.1110(2)	13.611(1)
**b(Å)**	16.1012(2)	16.1068(8)	16.1139(4)	16.0630(3)	26.1672(5)
**c(Å)**	13.2509(5)	13.228(1)	13.2366(5)	13.1703(2)	6.7381(5)
**β(°)**	132.240(6)	122.037(5)	122.113(2)	122.193(2)	136.14(1)
**Cell volume/Å^3^**	1,844.4(2)	1,837.3(2)	1,836.9(1)	1,810.17(7)	1,662.8(4)
**Z**	4	4	4	4	4
**Calc. density/mg·m^−3^**	1.987	2.003	2.012	2.063	1.974
**Absorption coefficient/mm^−1^**	4.110	4.37	4.628	5.227	4.29
**F(000)**	1,068	1,072	1,076	1,084	944
**Crystal size/mm^3^**	0.153 × 0.084 × 0.030	0.09 × 0.08 × 0.04	0.206 × 0.157 × 0.039	0.266 × 0.083 × 0.044	0.09 × 0.08 × 0.04
**Theta range for data collection/°**	3.27 to 31.81	3.28 to 31.16	3.27 to 31.26	3.29 to 31.39	3.3 to 31.4
**Index ranges**	−17≤ *h* ≤ 17, −23 ≤ *k* ≤ 22,	−13 ≤ *h* ≤ 14, −22 ≤ *k* ≤ 23,	−14 ≤ *h* ≤ 14, −23 ≤ *k* ≤ 23,	−14 ≤ *h* ≤ 14, −23 ≤ *k* ≤ 23,	−19 ≤ *h* ≤ 19, −38 ≤ *k* ≤ 37,
	−18 ≤ *l* ≤19	−18 ≤ *l* ≤19	−18 ≤ *l* ≤ 19	−18 ≤ *l* ≤ 19	−9 ≤ *l* ≤ 9
**Reflections collected**	22,135	12,103	15,702	10,974	12,022
**Independent reflections**	6,297	5,932	5,982	5,968	2,761
**Completeness to theta = 67°**	99.80%	99.04%	99.45%	99.80%	99.78%
**Absorption correction**	multi-scan	multi-scan	multi-scan	multi-scan	multi-scan
**Max. and min transmission**	0.933 and 0.883	0.836 and 0.678	0.836 and 0.424	1 and 0.861	0.838 and 0.682
**Refinement method**	Full-matrix least squares on *F*^2^	Full-matrix least squares on *F*^2^	Full-matrix least squares on *F*^2^	Full-matrix least squares on *F*^2^	Full-matrix least squares on *F*^2^
**Data/restraints/parameters**	5,851/2/286	5,365/15/280	5,465/6/286	5,366/6/280	2,548/0/119
**Goodness -of -fit on *F*^2^**	1.052	1.03	1.016	1.022	1.08
**Final *R* indices** **(*I* > 2sigma (*I*))**	*R*1 = 0.0288 (*wR*2 = 0.0682)	*R*1 = 0.055 (*wR*2 = 0.150)	*R*1 = 0.028 (*wR*2 = 0.061)	*R*1 = 0.0283 (*wR*2 = 0.057)	*R*1 = 0.0343 (*wR*2 = 0.0585)
***R*** **indices (all data)**	*R*1 = 0.053 (*wR*2 = 0.0589)	*R*1 = 0.109 (*wR*2 = 0.236)	*R*1 = 0.048 (*wR*2 = 0.072)	*R*1 = 0.061 (*wR*2 = 0.0449)	*R*1 = 0.0436 (*wR*2 = 0.0624)
**Largest diff. peak and hole/eÅ^−3^**	0.545 and −1.335	2.74 and −3.21	0.950 and −1.452	0.736 and −0.103	1.70 and −1.60
**CCDC no.**	1444552	1444554	1444553	1444556	1444555

## Supplementary Materials

The following are available online at www.mdpi.com/2073-4360/8/3/86/s1, Figure S1: SEM images of **MS3**(a,b); **MS7** (c); **MS7a** (d) and **MS7b** (e); Figure S2: SEM images and EDX spectra of **MS5** (a,c) and **MS6** (b,d); Figure S3: Perspective view of the asymmetric unit of **MS3**(a) and **MS7b** (b); Figure S4: TGA/DTG curves of **MS1**, **MS2**, **MS5** and **MS6**; Figure S5: The emission spectrum of **MS3** upon excitation at 370 nm. The inset shows the excitation spectrum detecting luminescence at 557 nm. Table S1: The results of EDX quantitative analysis (in atomic %) for **MS5** and **MS6**; Table S2: Selected bond lengths (BL/Å) and bond angles (BA/°) for **MS1–4** and **MS7b**; Table S3: Hydrogen-bond geometry (Å, °) for **MS3**.

## References

[1] Xue D.X., Belmabkhout Y., Shekhah O., Jiang H., Adil K., Cairns A.J., Eddaoudi M. (2015). Tunable Rare Earth fcu-MOF Platform: Access to Adsorption Kinetics Driven Gas/Vapor Separations via Pore Size Contraction. J. Am. Chem. Soc..

[2] Alezi D., Peedikakkal A.P., Weseliński Ł.J., Guillerm V., Belmabkhout Y., Cairns A.J., Chen Z., Wojtas Ł., Eddaoudi M. (2015). Quest for highly connected metal–organic framework platforms: Rare-Earth polynuclear clusters versatility meets net topology needs. J. Am. Chem. Soc..

[3] Kim H.K., Yun W.S., Kim M.-B., Kim J.Y., Bae Y.-S., Lee J., Jeong N.C. (2015). A Chemical route to activation of open metal sites in the copper-based metal–organic framework materials HKUST-1 and Cu-MOF-2. J. Am. Chem. Soc..

[4] Ma D.-Y., Li Z., Xiao J.-X., Deng R., Lin P.-F., Chen R.-Q., Liang Y.-Q., Guo H.-F., Liu B., Liu J.-Q. (2015). Hydrostable and nitryl/methyl-functionalized metal–organic framework for drug delivery and highly selective CO_2_ adsorption. Inorg. Chem..

[5] Howarth A.J., Katz M.J., Wang T.C., Platero-Prats A.E., Chapman K.W., Hupp J.T., Farha O.K. (2015). High Efficiency Adsorption and removal of selenate and selenite from water using metal–organic frameworks. J. Am. Chem. Soc..

[6] Van de Voorde B., Bueken B., Denayer J., de Vos D. (2014). Adsorptive separation on metal-organic frameworks in the liquid phase. Chem. Soc. Rev..

[7] Horcajada P., Gref R., Baati T., Allan P.K., Maurin G., Couvreur P., Férey G., Morris R.E., Serre C. (2012). Metal organic frameworks in biomedicine. Chem. Rev..

[8] Lammert M., Wharmby M.T., Smolders S., Bueken B., Lieb A., Lomachenko K.A., de Vos D., Stock N. (2015). Cerium-based metal organic frameworks with UiO-66 architecture: Synthesis, properties and redox catalytic activity. Chem. Commun..

[9] Valvekens P., Vandichel M., Waroquier M., Van Speybroeck V., de Vos D. (2014). Metal-dioxidoterephthalate MOFs of the MOF-74 type: Microporous basic catalysts with well-defined active sites. J. Catal..

[10] Vermoortele F., Bueken B., Le Bars G., van de Voorde B., Vandichel M., Houthoofd K., Vimont A., Daturi M., Waroquier M., Van Speybroeck V. (2013). Synthesis Modulation as a Tool To Increase the catalytic activity of metal-organic frameworks: The unique case of UiO-66(Zr). J. Am. Chem. Soc..

[11] Roy S., Chakraborty A., Maji T.K. (2014). Lanthanide–organic frameworks for gas storage and as magneto-luminescent materials. Coord. Chem. Rev..

[12] Abdelbaky M.S.M., Amghouz Z., García-Granda S., García J.R. (2014). A metal-organic framework assembled from Y(III), Li(I), and terephthalate: Hydrothermal synthesis, crystal structure, thermal decomposition and topological studies. Dalton Trans..

[13] Abdelbaky M.S.M., Amghouz Z., Fernández-Zapico E., García-Granda S., García J.R. (2015). Metal-organic Frameworks assembled from lanthanide and 2,5-pyridinedicaboxylate with cubane-like [Ln_4_(OH)_4_] building Units. J. Solid State Chem..

[14] Amghouz Z., García-Granda S., García J.R., Ferreira R.A. S., Mafra L., Carlos L.D., Rocha J. (2012). Series of metal organic frameworks assembled from Ln(III), Na(I), and Chiral flexible-achiral rigid dicarboxylates exhibiting tunable UV–vis–IR light emission. Inorg. Chem..

[15] Bag P.P., Wang X.-S., Cao R. (2015). Microwave-assisted large scale synthesis of lanthanide metal–organic frameworks (Ln-MOFs), having a preferred conformation and photoluminescence properties. Dalton Trans..

[16] Cui Y., Yue Y., Qian G., Chen B. (2012). Luminescent functional metal–organic frameworks. Chem. Rev..

[17] Kido J. (2002). Organo lanthanide metal complexes for electroluminescent materials. Chem. Rev..

[18] Min Z., Singh-Wilmot M.A., Cahill C.L., Andrews M., Taylor R. (2012). Isoreticular lanthanide metal-organic frameworks: Syntheses, structures and photoluminescence of a family of 3D phenylcarboxylates. Eur. J. Inorg. Chem..

[19] Liu Y., Zhang Y., Hu G.H., Zhou S., Fan R., Yang Y., Xu Y. (2015). A Series of lanthanide metal–organic frameworks with interesting adjustable photoluminescence constructed by helical chains. Chem. Eur. J..

[20] Decadt R., Hecke K.V., Depla D., Leus K., Weinberger D., Driessche I.V., van der Voort P., Deun R.V. (2012). Synthesis, crystal structures, and luminescence properties of carboxylate based rare-earth coordination polymers. Inorg. Chem..

[21] Rao X., Song T., Gao J., Cui Y., Yang Y., Wu C., Chen B., Qian G. (2013). A highly sensitive mixed lanthanide metal–organic framework self-calibrated luminescent thermometer. J. Am. Chem. Soc..

[22] Lee W.R., Ryu D.W., Lee J.W., Yoon J.H., Koh E.K., Hong C.S. (2010). Microporous lanthanide-organic frameworks with open metal sites: Unexpected sorption propensity and multifunctional properties. Inorg. Chem..

[23] Yang J., Song S.Y., Ma J.F., Liu Y.Y., Yu Z.T. (2011). Syntheses, structures, photoluminescence, and gas adsorption of rare earth–organic frameworks based on a flexible tricarboxylate. Cryst. Growth Des..

[24] Biswas S., Jena H.S., Goswami S., Sanda S., Konar S. (2014). Synthesis and characterization of two lanthanide (Gd^3+^ and Dy^3+^)-based three-dimensional metal organic frameworks with squashed metallomacrocycle type building blocks and their magnetic, sorption, and fluorescence properties study. Cryst. Growth Des..

[25] Nayak S., Nayek H.P., Pietzonka C., Novitchi G., Dehnen S. (2011). A series of three-dimensional lanthanide MOFs: Observation of reversible structural changes controlled by solvent desorption–adsorption, and magnetic properties. J. Mol. Struct..

[26] Silva P., Cunha-Silva L., Silva N.J. O., Rocha J., Almeida Paz F.A. (2013). Metal–organic frameworks assembled from erbium tetramers and 2,5-pyridinedicarboxylic acid. Cryst. Growth Des..

[27] Shi P.-F., Chen Z., Xiong G., Shen B., Sun J.-Z., Cheng P., Zhao B. (2012). Structures, luminescence, and magnetic properties of several three-dimensional lanthanide–organic frameworks comprising 4-carboxyphenoxy acetic acid. Cryst. Growth Des..

[28] Feng X., Zhao J., Liu B., Wang L., Ng S., Zhang G., Wang J., Shi X., Liu Y. (2010). A series of lanthanide−organic frameworks based on 2-propyl-1h-imidazole-4,5-dicarboxylate and oxalate: Syntheses, structures, luminescence, and magnetic properties. Cryst. Growth Des..

[29] Chen Q., Wang X.-F., Hu H.-M., Wang J., An R., Dong F.-X., Yang M.-L., Xue G.-L. (2014). Effect of pH on the construction of lead coordination polymers by the diverse coordination modes of sulfonate functionalized imidazophenanthroline derivative ligand. Polyhedron.

[30] Ma L.-F., Wang L.-Y., Lu D.-H., Batten S.R., Wang J.-G. (2009). Structural variation from 1D to 3D: Effects of temperature and pH Value on the construction of Co(II)-H_2_tbip/bpp mixed ligands system. Cryst. Growth Des..

[31] Gu J., Gao Z., Tang Y. (2012). pH and Auxiliary ligand influence on the structural variations of 5(2′-carboxylphenyl) nicotate coordination polymers. Cryst. Growth Des..

[32] Santra A., Bharadwaj P.K. (2014). Solvent-induced structural diversity of partially fluorinated, stable Pb(II) metal–organic frameworks and their luminescence properties. Cryst. Growth Des..

[33] Lu W., Wei Z., Gu Z.-Y., Liu T.-F., Park J., Park J., Tian J., Zhang M., Zhang Q., Gentle T. (2014). Tuning the structure and function of metal–organic frameworks via linker design. Chem. Soc. Rev..

[34] Yaghi O.M., O’Keeffe M., Ockwig N.W., Chae H.K., Eddaoudi M., Kim J. (2003). Reticular synthesis and the design of new materials. Nature.

[35] Chae H.K., Siberio-Perez D.Y., Kim J., Go Y., Eddaoudi M., Matzger A.J., O’Keeffe M., Yaghi O.M. (2004). A route to high surface area, porosity and inclusion of large molecules in crystals. Nature.

[36] Thuery P., Masci B. (2010). Two- and Three-dimensional assemblies formed by alkali metal (Li^+^−Cs^+^) and Ba^2+^ ions with Bicyclo[2.2.2]oct-7-ene-2,3,5,6-tetracarboxylic acid. Cryst. Growth Des..

[37] Horike S., Matsuda R., Tanaka D., Mizuno M., Endo K., Kitagawa S. (2006). Immobilization of sodium ions on the pore surface of a porous coordination polymer. J. Am. Chem. Soc..

[38] Hou L., Zhang J.-P., Chen X.-M., Ng S.W. (2008). Two highly-connected, chiral, porous coordination polymers featuring novel heptanuclear metal carboxylate clusters. Chem. Commun..

[39] Tominaka S., Yeung H.H.-M., Henke S., Cheetham A.K. (2016). Coordination environments and π-conjugation in dense lithium coordination polymers. CrystEngComm.

[40] Gou L., Zhang H.-X., Fan X.-Y., Li D.-L. (2013). Lithium based coordination polymer as anode for Li-ion battery. Inorg. Chim. Acta..

[41] Liu Y.-Y., Zhang J., Xu F., Sun L.-X., Zhang T., You W.-S., Zhao Y., Zeng J., Cao Z., Yang D. (2008). Lithium-based 3D coordination polymer with hydrophilic structure for sensing of solvent molecules. Cryst. Growth Des..

[42] Banerjee D., Kim S.J., Parise J.B. (2009). Lithium based metal−organic framework with exceptional stability. Cryst. Growth Des..

[43] Banerjee D., Borkowski L.A., Kim S.J., Parise J.B. (2009). Synthesis and Structural characterization of lithium-based metal−organic frameworks. Cryst. Growth Des..

[44] Banerjee D., Parise J.B. (2011). Recent advances in s-block metal carboxylate networks. Cryst. Growth Des..

[45] Peng G., Ma L., Cai J., Liang L., Deng H., Kostakis G.E. (2011). Influence of alkali metal cation (Li(I), Na(I), K(I)) on the construction of chiral and achiral heterometallic coordination polymers. Cryst. Growth Des..

[46] Frigoli M., El Osta R., Marrot J., Medina M.E., Walton R.I., Millange F. (2013). Heterobimetallic sodium–lithium based metal–organic framework showing the β-cristobalite topology and having high permanent porosity. Eur. J. Inorg. Chem..

[47] Mendoza-Cortes J.L., Han S.S., Goddard W.A. (2012). High H_2_ uptake in Li-, Na-, K-metalated covalent organic frameworks and metal organic frameworks at 298 K. J. Phys. Chem. A.

[48] Han S.S., Goddard W.A. (2007). Lithium-Doped Metal-organic frameworks for reversible H_2_ storage at ambient temperature. J. Am. Chem. Soc..

[49] Mulfort K.L., Hupp J.T. (2008). Alkali metal cation effects on hydrogen uptake and binding in metal-organic frameworks. Inorg. Chem..

[50] Blomqvist A., Araujo C.M., Srepusharawoot P., Ahuja R. (2007). Li-decorated metal–organic framework 5: A route to achieving a suitable hydrogen storage medium. Proc. Natl. Acad. Sci. USA.

[51] Dalach P., Frost H., Snurr R.Q., Ellis D.E. (2008). Enhanced hydrogen uptake and the electronic structure of lithium-doped metal-organic frameworks. J. Phys. Chem. C.

[52] Mavrandonakis A., Tylianakis E., Stubos A.K., Froudakis G.E. (2008). Why Li doping in MOFs enhances H_2_ storage capacity? A multi-scale theoretical study. J. Phys. Chem. C.

[53] Klontzas E., Mavrandonakis A., Tylianakis E., Froudakis G.E. (2008). Improving hydrogen storage capacity of MOF by functionalization of the organic linker with lithium atoms. Nano Lett..

[54] Kolmann S.J., Chan B., Jordan M.J.T. (2008). Modelling the interaction of molecular hydrogen with lithium-doped hydrogen storage materials. Chem. Phys. Lett..

[55] Wang Lu., Han Y., Feng X., Zhou J., Qi P., Wang B. (2016). Metal–organic frameworks for energy storage: Batteries and supercapacitors. Coord. Chem. Rev..

[56] Zou F., Chen Y.-M., Liu K., Yu Z., Liang W., Bhaway S.M., Gao M., Yu Z. (2016). Metal organic frameworks derived hierarchical hollow NiO/Ni/Graphene composites for lithium and sodium storage. ACS Nano.

[57] Kong S., Dai R., Li H., Sun W., Wang Y. (2015). Microwave hydrothermal synthesis of Ni-based metal−organic frameworks and their derived yolk−shell NiO for Li-ion storage and supported ammonia borane for hydrogen desorption. ACS Sustain. Chem. Eng..

[58] Schmidt S., Sheptyakov D., Jumas J.-C., Medarde M., Benedek P., Novák P., Sallard S., Villevieille C. (2015). Lithium iron methylenediphosphonate: A model material for neworganic−inorganic hybrid positive electrode materials for Li ion batteries. Chem. Mater..

[59] Amghouz Z., Roces L., García-Granda S., García J.R., Souhail B., Mafra L., Shi F.-N., Rocha J. (2010). Metal organic frameworks assembled from Y(III), Na(I), and chiral flexible-achiral rigid dicarboxylates. Inorg. Chem..

[60] Han Y., Li X., Li L., Ma C., Shen Z., Song Y., You X. (2010). Structures and properties of porous coordination polymers based on lanthanide carboxylate building units. Inorg. Chem..

[61] Black C.A., Costa J.S., Fu W.T., Massera C., Roubeau O., Teat S.J., Aromı G., Gamez P., Reedijk J. (2009). 3-D lanthanide metal-organic frameworks: Structure, photoluminescence, and magnetism. Inorg. Chem..

[62] Liu Y.-Y., Zhang J., Sun L.-X., Xu F., You W.-S., Zhao Y. (2008). Solvothermal synthesis and characterization of a lithium coordination polymer possessing a highly stable 3D network structure. Inorg. Chem. Commun..

[63] Stein I., Ruschewitz U. (2006). Poly[di-[mu]3-aqua-[mu]4-terephthalato-dirubidium]. Acta Crystallogr. Sect. E.

[64] Stein I., Ruschewitz U. (2007). Poly[di-[mu]3-aqua-[mu]4-terephthalato-dicaesium]. Acta Crystallogr. Sect. E.

[65] Dale S.H., Elsegood M.R.J. (2003). Poly[sodium(I)-[mu]6-hydrogen benzene-1,4-dicarboxylato]. Acta Crystallogr. Sect. C.

[66] (2008). CrysAlis CCD.

[67] (2008). CrysAlis RED.

[68] Sheldrick G.M. (1997). SHELXL-97, Program for Refinement of Crystal Structures.

[69] Parkin S., Moezzi B., Hope H. (1995). XABS2: An empirical absorption correction program. J. Appl. Crystallogr..

[70] Spek A.L. (1990). PLATON, an integrated tool for the analysis of the results of a single crystal structure determination. Acta Crystallogr. Sect. A Fundam. Crystallogr..

[71] Spek A.L. (1998). PLATON, a multipurpose crystallographic tool.

[72] Brandenburg K. (2007). DIAMOND.

[73] Blatov V.A. Multipurpose crystallochemical analysis with the program package TOPOS. http://www.topos.ssu.samara.ru.

[74] Blatov V.A. (2012). Nanocluster analysis of intermetallic structures with the program package TOPOS. Struct. Chem..

[75] Dieke G.H. (1968). Spectra and Energy Levels of Rare Earth Ionsin Crystals.

[76] Debasu M.L., Ananias D., Rocha J., Malta O.L., Carlos L.D. (2013). Energy-transfer from Gd(III) to Tb(III) in (Gd,Yb,Tb)PO_4_ nanocrystals. Phys. Chem. Chem. Phys..

[77] Li H., Zhu G., Ren H., Li Y., Hewitt I.J., Qiu S. (2008). The synthesis of multiwalled rare-earth phosphate nanomaterials using organophosphates with upconversion properties. Eur. J. Inorg. Chem..

