# Supplementary material for: Synthesis, Structures and Luminescence Properties of Metal-Organic Frameworks Based on Lithium-Lanthanide and Terephthalate
Source: Polymers (Basel). 2016 Mar 16;8(3):86. doi: 10.3390/polym8030086 (PMC6432550; doi:10.3390/polym8030086)
Supplement: Supplementary file 1 [file polymers-08-00086-s001.pdf]

# Supplementary Materials: Synthesis, Structures, and Luminescence Properties of Metal Organic Frameworks Based on Lithium-Lanthanide and Terephthalate

**Mohammed S. M. Abdelbaky, Zakariae Amghouz, Santiago García-Granda and José R. García**

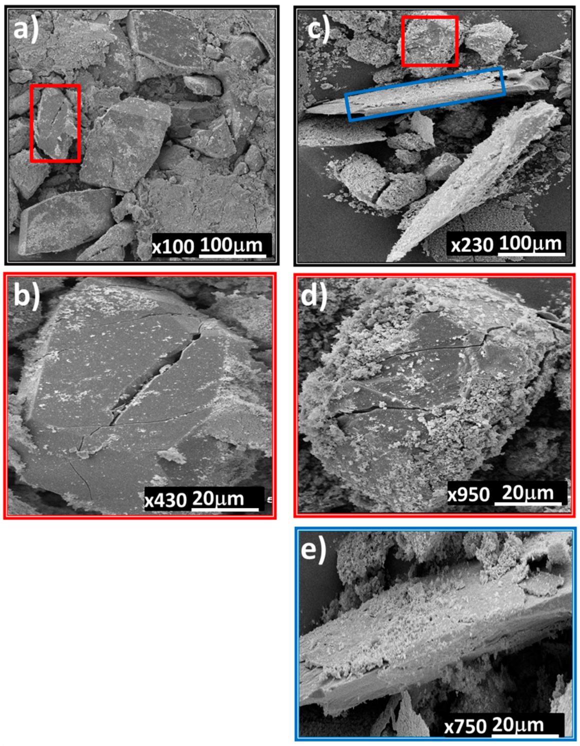


**Figure S1.** SEM images of **MS3** (**a**,**b**); **MS7** (**c**); **MS7a** (**d**) and **MS7b** (**e**).

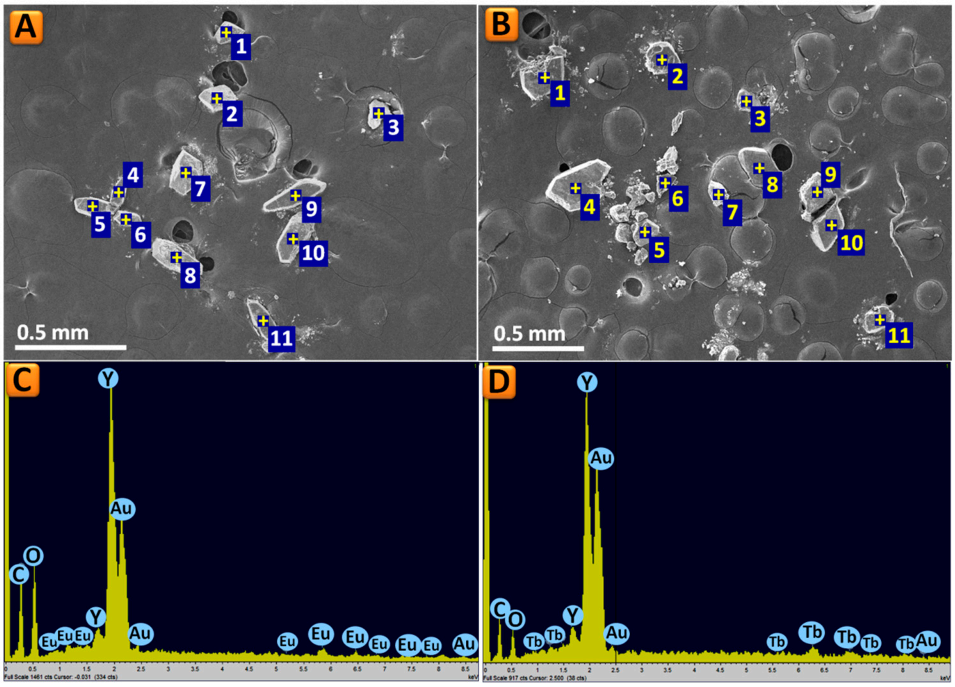


**Figure S2.** SEM images and EDX spectra of **MS5** (**A**,**C**) and **MS6** (**B**,**D**).

**Table S1.** The results of EDX quantitative analysis (in atomic %) for **MS5** and **MS6**.

| Spectrum | MS5 | | MS6 | |
|---|---|---|---|---|
| | **Y** | **Eu** | **Y** | **Tb** |
| **1** | 94.5 | 5.5 | 97.3 | 2.7 |
| **2** | 96.7 | 3.3 | 74.7 | 25.3 |
| **3** | 96.5 | 3.5 | 97.9 | 2.1 |
| **4** | 95.5 | 4.5 | 96.9 | 3.1 |
| **5** | 97.6 | 2.4 | 96.3 | 3.7 |
| **6** | 95.7 | 4.3 | 96.6 | 3.4 |
| **7** | 94.7 | 5.3 | 93.5 | 6.5 |
| **8** | 95.0 | 5.0 | 94.9 | 5.1 |
| **9** | 96.4 | 3.6 | 95.2 | 4.8 |
| **10** | 96.6 | 3.4 | 92.2 | 7.8 |
| **11** | 96.6 | 3.4 | 93.1 | 6.9 |
| **Mean** | 96.0 | 4.0 | 94.0 | 6.4 |
| **Std. deviation** | 0.9 | 0.9 | 6.5 | 6.5 |
| **Max.** | 97.6 | 5.4 | 97.9 | 25.3 |
| **Min.** | 94.5 | 2.4 | 74.68 | 2.1 |

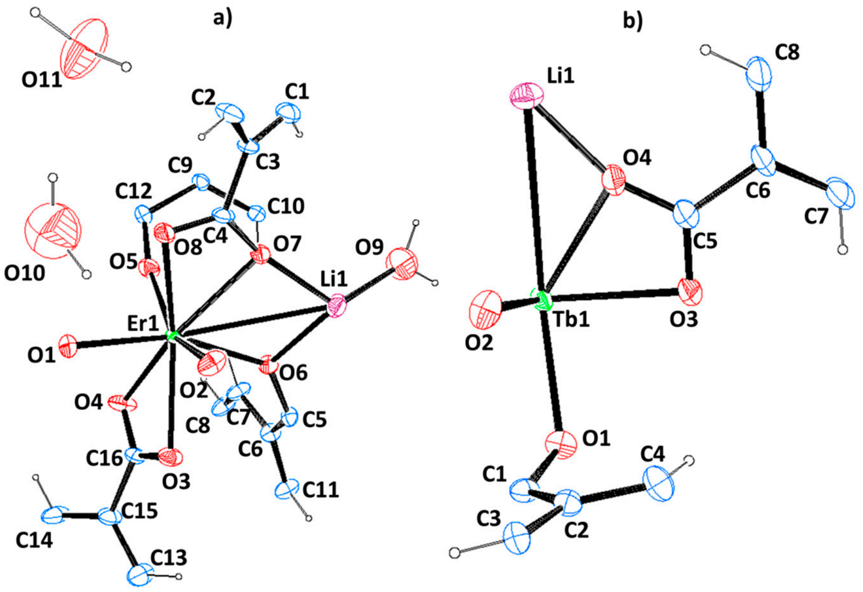


**Figure S3.** Perspective view of the asymmetric unit of **MS3** (**a**) and **MS7b** (**b**).

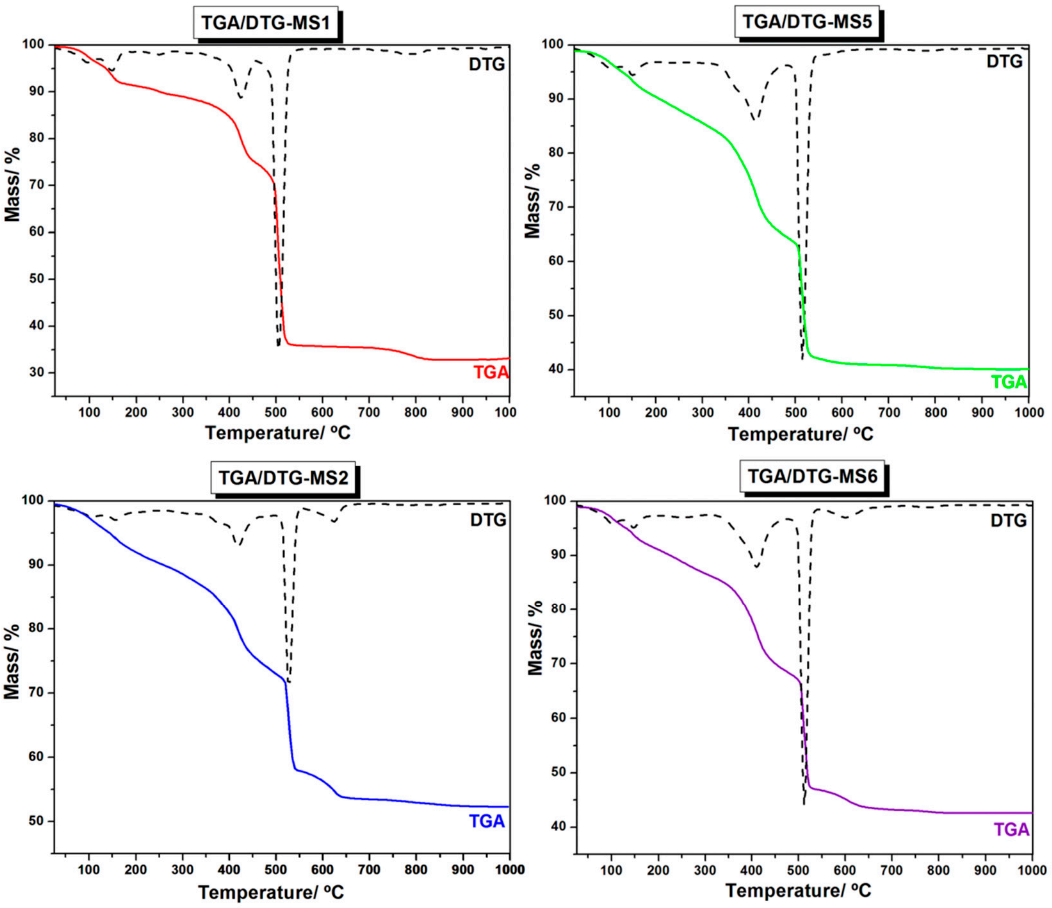


**Figure S4.** TGA/DTG curves of **MS1**, **MS2**, **MS5** and **MS6**.

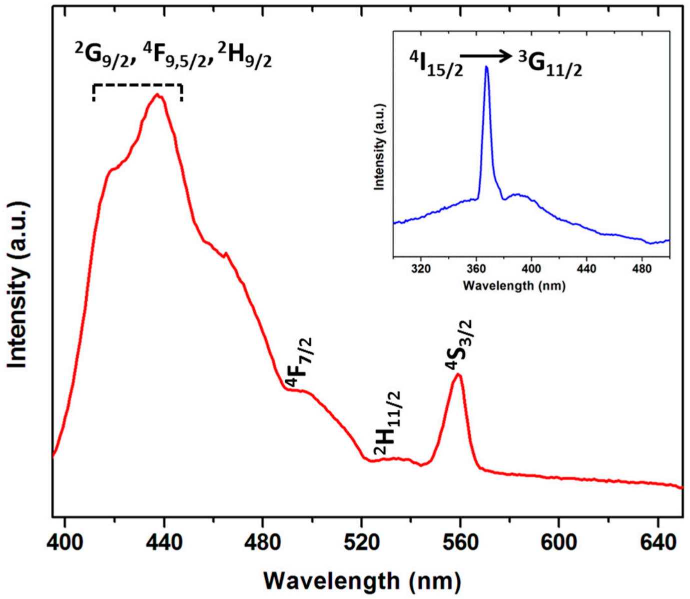


**Figure S5**. The emission spectrum of **MS3** upon excitation at 370 nm. The inset shows the excitation spectrum detecting luminescence at 557 nm.

**Table S2.** Selected bond lengths(BL/Å) and bond angles(BA/°) for **MS1–4** and **MS7b**.

| MS1 | BL/BA | MS2 | BL/BA | MS3 | BL/BA | MS4 | BL/BA | MS7b | BL/BA |
|---|---|---|---|---|---|---|---|---|---|
| Dy1–O1 | 2.426(2) | Ho1–O1 | 2.419(6) | Er1–O1 | 2.274(2) | Yb1–O1 | 2.407(2) | Tb1–O1 | 2.394(2) |
| Dy1–O2 | 2.473(2) | Ho1–O2 | 2.469(6) | Er1–O2 | 2.294(2) | Yb1–O2 | 2.424(2) | Tb1–O2 | 2.279(2) |
| Dy1–O3 | 2.350(2) | Ho1–O3 | 2.302(5) | Er1–O3 | 2.455(2) | Yb1–O3 | 2.384(3) | Tb1–O3 | 2.410(2) |
| Dy1–O4 | 2.311(2) | Ho1–O4 | 2.335(5) | Er1–O4 | 2.412(2) | Yb1–O4 | 2.430(2) | Tb1–O4 | 2.434(3) |
| Dy1–O5 | 2.263(2) | Ho1–O5 | 2.445(6) | Er1–O5 | 2.241(2) | Yb1–O5 | 2.296(2) | Li1–O1 | 1.997(4) |
| Dy1–O6 | 2.294(2) | Ho1–O6 | 2.439(6) | Er1–O6 | 2.325(2) | Yb1–O6 | 2.270(3) | Li1–O3 | 1.888(5) |
| Dy1–O7 | 2.463(2) | Ho1–O7 | 2.274(5) | Er1–O7 | 2.447(2) | Yb1–O7 | 2.212(2) | Tb1–Li1 | 3.376(6) |
| Dy1–O8 | 2.445(2) | Ho1–O8 | 2.254(6) | Er1–O8 | 2.428(2) | Yb1–O8 | 2.245(3) | | |
| Li1–O2 | 1.926(6) | Li1–O2 | 1.900(2) | Li1–O3 | 1.918(6) | Li1–O2 | 1.925(7) | | |
| Li1–O3 | 2.055(5) | Li1–O4 | 2.060(2) | Li1–O6 | 2.050(6) | Li1–O4 | 1.919(7) | | |
| Li1–O7 | 1.927(6) | Li1–O5 | 1.960(2) | Li1–O7 | 1.937(5) | Li1–O5 | 2.052(7) | | |
| Li1–O1W | 1.894(6) | Li1–O9 | 1.880(2) | Li1–O9 | 1.892(6) | Li1–O1W | 1.882(8) | | |
| Dy1–Li1 | 3.405(5) | Ho1–Li1 | 3.410(2) | Er1–Li1 | 3.390(6) | Yb1–Li1 | 3.373(7) | | |
| O1–Dy1–O2 | 52.97(7) | O1–Ho1–O2 | 53.1(2) | O1–Er1–O2 | 86.66(9) | O1–Yb1–O2 | 53.89 (8) | O1–Tb1–O2 | 77.25(8) |
| O1–Dy1–O3 | 85.87(7) | O1–Ho1–O3 | 128.1(2) | O1–Er1–O3 | 83.51(8) | O1–Yb1–O3 | 145.15(9) | O1–Tb1–O3 | 126.87(9) |
| O1–Dy1–O4 | 128.30(7) | O1–Ho1–O4 | 85.4(2) | O1–Er1–O4 | 77.62(8) | O1–Yb1–O4 | 146.1 (1) | O1–Yb1–O4 | 82.86(9) |
| O1–Dy1–O5 | 77.64(7) | O1–Ho1–O5 | 144.9(2) | O1–Er1–O5 | 96.52(8) | O1–Yb1–O5 | 122.19(9) | O1–Li1–O5 | 122.19(9) |
| O1–Dy1–O6 | 77.73(8) | O1–Ho1–O6 | 145.8(2) | O1–Er1–O6 | 159.21(8) | O1–Yb1–O6 | 74.03(9) | O1–Li1–O3 | 115.3(1) |
| O1–Dy1–O7 | 145.21(7) | O1–Ho1–O7 | 78.3(2) | O1–Er1–O7 | 132.13(7) | O1–Yb1–O7 | 80.48(9) | | |
| O1–Dy1–O8 | 145.92(8) | O1–Ho1–O8 | 77.6(2) | O1–Er1–O8 | 78.85(8) | O1–Yb1–O8 | 78.46(9) | | |
| O2–Li1–O7 | 114.6(3) | O2–Li1–O5 | 114.5(7) | O3–Li1–O7 | 114.9(3) | O2–Li1–O4 | 115.4(4) | | |
| O2–Li1–O1W | 110.1(3) | O2–Li1–O9 | 110.8(8) | O3–Li1–O9 | 110.2(3) | O2–Li1–OW | 118.7(3) | | |

**Table S3.** Hydrogen-bond geometry (Å, °) for **MS3**.

| **D–H···A** | **D–H** | **H···A** | **D···A** | **D–H···A** |
|---|---|---|---|---|
| O9–H1W···O11 [I] | 0.89 | 1.96 | 2.835 | 167 |
| O9–H2W···O10 [II] | 0.91 | 1.92 | 2.819 | 172 |
| O10–H4W···O11 [III] | 0.81 | 2.17 | 2.967 | 169 |
| O11–H5W···O8 [IV] | 0.89 | 1.87 | 2.673 | 178 |
| O11–H6W···O4 [V] | 0.87 | 2.13 | 2.847 | 139 |

Symmetry codes: (I) x,-y+1/2+1,+z-1/2; (II) x,+y+1,+z; (III) x,y,z; (IV) -x+1,-y+1,-z+1; (V) x+1,+y-1,+z+1.

© 2016 by the authors; licensee MDPI, Basel, Switzerland. This article is an open access article distributed under the terms and conditions of the Creative Commons by Attribution (CC-BY) license (http://creativecommons.org/licenses/by/4.0/).
